# Outcome and Determining Characteristics of ICU Patients with Acute Kidney Injury in a Low-Income Country, a Multicenter Experience

**DOI:** 10.2478/jccm-2024-0037

**Published:** 2024-10-31

**Authors:** Abubakar Ballah, Jika Mohammed, Gyadale Abdulhamid Njidda, Halima Bidemi Yahaya, Nwokorie Mabong Rosemary, Ibrahim Naziru, Adamu Yusuf Baffah, Hassan Ali Maina, Hajara Galadima, Abdullahi Maryam Mohammed, Franklin Andibanbang, Adam Ibrahim Abdullahi

**Affiliations:** Abubakar Tafawa Balewa University, Bauchi, Nigeria; Federal Medical Centre Gombe, Gombe, Nigeria; Airdale NHS Foundation Trust, Steeton, England; Nile University of Nigeria, Abuja, Nigeria; Modibbo Adama University of Technology, Yola, Nigeria; Aminu Kano Teaching Hospital, Kano, Nigeria; Saudi Arabia Ministry of Health, Fayfa, Saudi Arabia; Bauchi State University Gadau, Bauchi, Nigeria

**Keywords:** acute kidney injury, intensive care unit, mortality, KDIGO stratification, APACHE I

## Abstract

**Background:**

Acute kidney injury (AKI) is a disease that affects millions of people globally making it a major public health concern. It is defined as an abrupt decrease in kidney function that occurs within ours affecting both the structure and functionality of the kidneys.

The outcome of AKI and the determinants in Nigeria are largely unknown. This study aimed to describe the determining factors of the outcome of AKI patients admitted into the ICU of three tertiary health institutions in Northeast Nigeria.

**Methods:**

The study is a prospective multicentered observational study of the patients admitted into the ICU in three tertiary health institutions from January 2022 to December 2023. KDIGO criteria was used to define AKI. The outcome of the study was to determine survivors among the patients admitted into the ICU with AKI or developed AKI while in ICU and also the determinants of mortality. A chi-square test was done to determine the association between the dependent variable (patient outcome) and the independent variables. To determine the predictors of patient outcomes, a regression analysis was done. The sociodemographic data of the patients admitted during these periods were studied in addition to Acute Physiology and Chronic Health Evaluation (APACHE) II, Kidney Disease: Improving Global Outcomes (KDIGO), Average length of stay in the ICU, Admitting/referring ward (Obstetrics, Gynae, Medical, Surgical or Emergency unit), Ability to afford care (out of pocket payment, social welfare or through Health insurance Scheme, Co-morbidity (presence or absence of comorbidity), Interventions done while in ICU (use of vasopressors and inotropes, mechanical ventilation (MV) support and renal replacement therapy (RRT) and outcome (discharge to the wards or mortality).

**Results:**

Of 1494 patient records screened, 464 met the inclusion criteria. The overall incidence of AKI was 57%. About 53% were females, the mean age was 42.2 years, and 81% of the patients had a normal BMI (18.5 – 24.9). About 40% of the patients had APACHE II scores ≥ 29%. More than three-quarters (79.5%) of the patients paid for their health care expenditure out-of-pocket. Most patients (72%) were from the Medical and Gynae/Ward. Mortality was highest (54.2%) among patients who were brought into the ICU from the Medical ward. Most patients admitted were KDIGO I (44.3%) followed by KDIGO II (35.1%). Among the patients, 61.2% present with one or more comorbidity. Mortality was higher (50%) among those with comorbidity compared to 13.6% among those without comorbidity. Mortality was lowest among patients who stayed in the ICU between 8–14 days compared to those who stayed > 2 weeks. Most of the patients (72%) were from the Medical and Gynae/Ward. Mortality was highest (54.2%) among patients who were brought into the ICU from the Medical ward followed by those brought in from the Obstetric and Gynecological ward (20.4%). An association was found between the intervention received in the ICU and the outcome, which was found to be statistically significant (p < 0.001). A regression analysis was done to determine the predictors of patients’ outcomes admitted in the ICU. The results showed that APACHE II score greater than 10 (p-value < 0.001), presence of comorbidities (p = 0.031) and intervention which included a combination of Vasopressors, mechanical ventilation and RRT (p < 0.01) are the predictors of patients’ outcome. The regression model is valid (X^2^ = 469.894, df = 24, p < 0.001) and it fits the sample as shown by the Hosmer and Lemeshow test (X^2^ = 7.749, p = 0.45, df = 8,). It also shows that the predictors account for 92% of patients’ outcomes (Nagelkerke R^2^ = 0.92).

**Conclusions:**

Our study revealed that the presence of comorbidity, high APACHE II score, and the need for interventional supports including both mechanical ventilatory and ionotropic, were found to be strong mortality predictors in patients with AKI.

## Introduction

Acute kidney injury (AKI) is a disease that affects millions of people globally making it a major public health concern. It is defined as an abrupt decrease in kidney function that occurs within ours affecting both the structure and functionality of the kidneys [1]. Acute kidney injury (AKI) has been defined by a consensus of scholars to include AKI of all severities and also to allow for study comparisons. The consensus definitions include the RIFLE classification. This was modified later on to the AKIN and the KDIGO criteria [2]. Globally, over 13 million patients are affected, the majority of whom live in low-income and middle-income countries (LMICs). About 1.5 million deaths due to AKI occur annually globally [3].

AKI is well known as a common complication among patients admitted into the ICU and is found to be associated with poor outcomes including an increase in the duration of hospital and ICU stay, progression to chronic kidney disease (CKD), and an increased mortality risk [4]. There are numerous possible etiologic factors for AKI in the critically ill patient population including eclampsia, sepsis, states of low cardiac output, major surgery, and nephrotoxic agents [5]. The incidence of AKI widely varies ranging from as low as 5% to as high as 60%, especially in the LMICs, and mortality can be as high as 80% in patients who require dialysis. Other factors influencing patient survival in AKI include the severity of underlying illness, age of the patient, the number and severity of coexisting illnesses, metabolic complications, as well as systemic life-threatening complications such as cardiac arrhythmia, myocardial infarction, pulmonary embolism, etc [6].

This study aims to determine the outcome and its determining characteristics of ICU patients with acute kidney injury.

## Methods

### Study design

In this prospective multicentered observational study, we reviewed 1496 patients admitted into the ICU of 3 tertiary hospitals across Northeast Nigeria from 1^st^ January 2022 to 31^st^ December 2023. Inclusion criteria were patients aged ≥ 18 years and diagnosed with AKI at admission or those who developed AKI while on admission were included. In comparison, exclusion criteria included aged < 18 years, patients with Chronic or end-stage renal disease or any terminal disease, AKI due to obstructive uropathy, severe co-morbidities, or already on renal replacement therapy. All the patients with laboratory definitions of Acute kidney Injury based on the KDIGO stratification (Increase in serum creatinine by ≥0.3 mg/dL (≥26.5 μmol/L) within 48 h; or increase in serum creatinine to ≥1.5 times baseline, which is known or presumed to have occurred within the prior seven days; or urine volume < 0.5 mL/kg/h for 6 h) and were admitted into the ICU of the participating hospitals were selected. Those who were initially admitted for a different pathology but later developed AKI while on admission and required ICU admission were also chosen for our study. Participating patients were reviewed daily by the intensivist who performs daily examinations, reviews investigation results, and institutes or reviews treatment protocol. We recorded all comorbidities and presented them as a table based on the proportion of the respondents with comorbidities. Intermittent dialysis was the dialyzing modality of choice and was indicated by creatinine ≥ 600 mmol/L, urea ≥ 30 mmol/L, severe metabolic acidosis, hyperkalemia, uremic pericarditis, uremic encephalopathy, and fluid overload. If mean arterial pressure falls below 60mmHg, we used norepinephrine at a dose of 0 to 0.04 μg kg^−1^ min^−1^, dopamine 0.3–0.5 μg kg^−1^ min^−1^, or phenylephrine 0.5 to 6 mcg/kg/min as vasopressors.

### Variables

The following variables were collected from the patient’s record: Age; Gender; Body Mass Index Acute Physiology and Chronic Health Evaluation (APACHE) II, Kidney Disease: Improving Global Outcomes (KDIGO), Average length of stay in the ICU, Admitting/referring ward (Obstetrics, Gynae, Medical, Surgical or Emergency unit), Ability to afford care (out of pocket payment, social welfare or through Health insurance Scheme, Co-morbidity (presence or absence of comorbidity), Interventions done while in ICU (use of vasopressors and inotropes, mechanical ventilation (MV) support and renal replacement therapy (RRT) and outcome (discharge to the wards or mortality).

### APACHE II interpretation

Minimum 0 and maximum 71; higher APACHE II score is associated with a higher risk of death. 0–9 is mild disease severity with a mortality of 4–8%, 10–19 moderate disease severity with a mortality of 15–24%, and ≥ 20 is severe disease mortality ranges between 40–85%.

### Statistical analysis

Descriptive statistical analysis results were presented as proportions and percentages for categorical variables and mean and standard deviation for continuous variables. The chi-square test was used to determine the association between the independent and the dependent variables. A regression analysis was used to determine the predictors of patients’ outcomes. A *p-value* of < 0.05 was considered statistically significant.

## Results

Three ICUs were used for the study all located in Northeast Nigeria. A total of 464 patients diagnosed with AKI either on admission or while in the ICU over 24 months were included in the study. The mean age was 42.2 years. 72.8% of the patients were aged less than 50. Mortality was significantly higher among those aged greater than 50 (45.2%). A chi-square analysis found a statistically significant relationship between age and outcome of patients (X^2^ = 26.98, df = 1, p < 0.01) as shown in Table 1.

**Table 1.** Association between independent variables and outcome

**Variables**	**Chi-square**	**df**	**P-value**
Age	26.980	1	0.001
Gender	0.150	1	0.698
BMI	95.265	2	< 0.001
APACHE	350.519	2	< 0.001
KDIGO class	93.712	3	< 0.001
Admitting ward	93.009	3	< 0.001
Co-morbidities	72.626	1	< 0.001
Source of AKI	0.074	1	0.785
Length of stay	16.588	2	< 0.001
Interventions	69.377	6	< 0.001
Bill payment	31.988	2	< 0.001

Clinical data of sampled patients indicating demographic profile (Age, Gender, BMI) clinical state (APACHE & KDIGO), co-morbidity, source of AKI (hospital-acquired or community-acquired), length of ICU stay, intervention rendered while in ICU (renal replacement therapy RRT, Vasopressors, Mechanical ventilation MV), bill payment (either out of pocket expenses, through the National health insurance scheme or the Social welfare services)

More than half (53%) of the patients were females. There was no significant difference in mortality among the sexes (X^2^ = 0.150, df = 1, p = 0.698).

Most of the patients (81%) had normal BMI with only 7% of them being overweight. Chi-square analysis revealed a statistically significant relationship between obesity and mortality (X^2^ = 95.265, df =2, p < 0.01).

About 40% of the patients had APACHE II scores ≥ 29% and mortality was highest in this group of patients (98%). Further, Chi-square analysis revealed a statistically significant relationship between this group of APACHE II patients and outcome (X^2^ = 350.519, df = 2, p< 0.01).

More than three-quarters (79.5%) of the patients paid for their health care expenditure out-of-pocket while the rest either paid through the National Health Insurance Scheme (11%) or the social welfare team of the hospitals (9%). Mortality was found to be higher (60%) among patients who were supported by the social welfare team of the hospital. A statistically significant relationship was also found between patients who were supported by the social welfare team and outcomes (X^2^ = 31.988, df = 2, p < 0.01).

Most of the patients (72%) were from the Medical and Gynae/Ward. Mortality was highest (54.2%) among patients who were brought into the ICU from the Medical ward followed by those brought in from the Obstetric and Gynecological ward (20.4%). The lowest mortality was reported among patients brought in from the Surgical ward (1.1%). A statistically significant relationship was found between the patients admitted from the medical ward and poor outcomes (X^2^ = 93.009, df = 3, p < 0.01).

Most of the patients admitted were KDIGO I (44.3%) followed by KDIGO II (35.1%). Mortality was highest among KDIGO III (64.9%) followed by KDIGO II (27%). A statistically significant relationship was found between KDIGO III and poor outcome (X^2^ = 93.712, df = 3, p < 0.01).

More than half of the patients (54%) acquired AKI while on admission. Mortality was similar among those admitted with AKI (27%) and those who developed AKI while on admission (28%). There is no statistically significant difference in the outcome between those who acquire AKI in the hospital and those who acquire it in the community (X^2^ = 0.074, df = 1, p < 0.785).

Among the patients, 61.2% present with one or more comorbidity. Mortality was higher (50%) among those with comorbidity compared to 13.6% among those without comorbidity. A statistically significant association was found between the presence of comorbidity and outcome (X^2^ = 72.626, df = 1, p < 0.001).

Mortality was lowest among patients who stayed in the ICU between 8–14 days compared to those who stayed > 2 weeks. A significant association was found between longer duration of stay in the ICU and outcome (X^2^ = 16.588, df = 2, p < 0.001). Mortality was highest (47.2%) among patients who required mechanical ventilation, renal replacement therapy, and vasopressors followed by 24.3% among patients who required renal replacement therapy and Inotropes, and lowest in those who required RRT alone. A statistically significant association (p < 0.001) was found between the intervention received in ICU and the outcome.

A regression analysis was done to determine the predictors of patients’ outcomes admitted in the ICU. Above 50 years, Female, Obesity, APACHE II ≥ 20, social welfare, emergency ward, KDIGO III, more than 14 days, and Mechanical ventilation + Vasopressors were used as references for Age, gender, BMI, APACHE II, method of payment of hospital bill, admitting ward, KDIGO, length of ICU stay and intervention respectively. The results showed that APACHE II score greater than 10 (p-value < 0.001), presence of comorbidities (p = 0.031) and intervention which included a combination of Vasopressors, mechanical ventilation and RRT (p < 0.01) are the predictors of patients’ outcome as shown in Table 2. The regression model is valid (X^2^ = 469.894, df = 24, p < 0.001) and it fits the sample as shown by the Hosmer and Lemeshow goodness of fit test (X^2^ = 7.749, df = 8, p = 0.458). It also shows that the predictors account for 92% of patients’ outcomes (Nagelkerke R^2^ = 0.92).

**Table 2.** Logistic regression table

**Variables**	**Odd ratio**	**95% CI lower**	**95% CI upper**	**p-value**
**Age**				
< 50 years	0.550	0.140	2.165	0.392
> 50 years		-	-	-

**Gender**				
Male	0.746	0.029	19.389	0.782
Female	-	-	-	-

**BMI**				
Underweight	0.746	0.029	19.386	0.860
Normal weight	0.000	0.000	-	0.997
Obese	-	-	-	-

**APACHEII**				
Mild	0.032	0.001	0.757	0.033
Moderate	-	0.000	0.002	< 0.001
Severe	-	-	-	-

**Classification**				
KDIGO1	9.087	0.558	148.023	0.121
KDIGO 2	1.144	0.060	21.693	0.929
KDIGO 3	-	-	-	-

**Source of AKI**				
Community	12.031	0.102	2.587	0.420
Hospital	-	-	-	-

**Admitting ward**				
Obs&Gyn	0.143	0.007	2.917	0.206
Medical	-	0.000	-	0.997
Surgical	0.133	0.006	2.899	0.199
Emergency	-	-	-	-

**Length of stay**				
≤ 7 days	0.249	0.007	8.716	0.444
8-14 days	0.000	0.000	-	0.999
> 14 days	-	-	-	-

**Comorbidities**				
Yes	12.031	0.031	0.102	2.587
No	-	-	-	-

**Interventions**				
RRT	0.000	0.000	-	0.995
Vasopressors	3.305	0.007	1501.012	0.702
MV	1.115	0.061	20.263	0.941
RRT + Vasopressors	1.403	0.109	18.047	0.795
RRT + MV	1.546	0.197	12.144	0.678
RRT + Vasopressors + MV	0.001	0.000	0.059	< 0.001
RRT + Vasopressors	-	-	-	-

**Bill payment source**				
Out of pocket	7.050	0.009	5554.736	0.566
Insurance scheme	2.972	0.147	59.937	0.477
Social welfare	-	-	-	-

Logistic regression analysis to determine the predictors of patients’ outcomes admitted in the ICU

## Discussion

In this prospective multicenter study, we conducted a study in three (3) ICUs on 464 patients to determine the outcome of AKI and its determining characteristics. To our knowledge, no similar study has been conducted before in Nigeria. We characterized the patients based on their age, gender, body mass index, APACHE II, KDIGO stratification, admitting/referring ward, ability to afford care, presence or absence of comorbidity, ICU length of stay, whether AKI was acquired while on admission in the participating hospitals or it was acquired in the community, Interventions done on the patient while in the ICU (use of vasopressors and inotropes, mechanical ventilation (MV) support and renal replacement therapy (RRT) and outcome (discharged back to the wards or mortality). In all the participating hospitals, patients admitted into the ICU are not discharged home directly, they are transferred back to the admitting wards if the condition improves based on protocol.

In our study, the mean age of patients with AKI was 42.2 years and mortality was found to be significantly higher among the elderly patients. Aging is regarded as an important risk factor for AKI. Older patients have reduced renal reserve due to decrease in functioning nephrons, decrease glomerular filtration rate, decreased renal blow and also changes in tubular function. Our finding is similar to a study reported by Hoste et al. who reported that increasing age is associated with significant mortality rates, ranging from 20% to 80%. Bucuvic et al reported in their research that mortality was over 65% in patients aged > 60 years. Studies by Mataloun et al, Pedersen et al and Martensson et al all-reported mortality rates ranging between 50% and 76.2% in the elderly [7,8,9]. Even though our results reveal similar findings to the studies mentioned earlier, we used different age groups in our study as we classified the age groups into > 50 or < 50 [5].

Mortality was also found to be significantly higher among obese patients with a BMI ≥ to 30. Obesity has been linked to prolonged mechanical ventilation, increased stay in the ICU, and increased morbidity and mortality among critically ill patients. It has been recognized as a risk factor for developing AKI in the ICU and also a risk factor for readmission following discharge. Obesity is increasingly recognized as a significant risk factor for AKI. It increases the risk of AKI through a complex interplay of metabolic, hemodynamic, inflammatory, and oxidative stress mechanisms. Obesity leads to various metabolic and hemodynamic alterations that increase the risk of AKI. It is known to increase renal blood flow and glomerular filtration rate (GFR) to compensate for the higher metabolic demands. This hyperfiltration can lead to glomerular hypertension, causing damage to the glomeruli over time. Obese and overweight patients commonly have insulin resistance and hyperglycemia which contributes to kidney damage by promoting inflammation, oxidative stress, and advanced glycation end-products (AGEs) accumulation. As a pro-inflammatory state, Obesity is characterized by elevated levels of inflammatory cytokines such as TNF-alpha, IL-6, and CRP. These inflammatory mediators can cause direct kidney injury. Other mechanisms that relate obesity to AKI include but are not limited to ectopic fat deposition, including in the kidneys, known as renal lipotoxicity, and the association of obesity with hypertension and diabetes. High blood pressure damages the renal arteries, reducing the blood supply to the kidneys and impairing their function while Hyperglycemia and insulin resistance can cause diabetic nephropathy, making the kidneys more susceptible to acute injury [8, 10].

Mortality has been established to be high among patients with obesity who developed AKI. The risk of death is higher in critically ill patients who are obese and develop AKI.

Some studies that looked at the outcomes of obese ICU patients with AKI show either a reduction in the mortality rate among them or showed no relationship between Obesity in the critically ill and elevated death [10–11].

Our study revealed that only 2% of the patients with APACHE scores ≥ 29 survived in the ICU. APACHE II is a good scoring system with good calibration and discrimination if applied to patients admitted into the ICU. It is a good mortality predictor among such patients. Studies by Bhadade et al., Maher et al., and Abosaif et al. also showed that the APACHE II scores were significantly higher in the dead patients compared to those discharged to the wards or home [12,13,14,15].

The KDIGO AKI guidelines were developed to regularize the AKI criteria based on the output of urine and the serum creatinine. We stratified our patients into 3 groups and Patients with AKI according to the KDIGO criteria 3 had a higher mortality than patients in KDIGO 1 and 2. KDIGO definition is associated with a progressively increased risk of long-term mortality in these patients [16].

In our study, the need for ionotropic support, Mechanical ventilation, and renal replacement therapy in the form of dialysis were found to be very strong predictors of death among AKI patients (p-value <0.001). Korula et al, found that the use of vasopressors (noradrenaline, dopamine or Phenylephrine) was an independent predictor of death in patients with AKI while the need to have mechanical ventilatory support also showed a significant relation with mortality in their study [17,18,19,20,21].

Our findings show mortality was higher among those who stayed for longer duration in the ICU more than two weeks [22, 23]. The commonest comorbidities among the patients were diabetes and hypertension. This is similar to the results of a study by Mosenye et al [24, 25]. Other comorbidities include ischemic heart disease and chronic liver disease.

### Limitations

Our study has some limitations like every other study from a low-resource setting. All the participating ICUs have no dialysis machine within the intensive care and critically ill patients have to be transported to another unit within the hospital for dialysis. If the patients cannot be transported due to their health condition or absence of a transport ventilator, such patients are managed conservatively with the use of Inotropes, nutritional support, and close monitoring until they improve to warrant transportation to the dialysis unit, or such patients expire. This may have affected the outcome under study which is mortality. We can’t measure PiCCO, CO as we are a low-resource setting. We depend solely on the basic hemodynamic monitoring (HR, NIBP, temperature, ECG, SPO_2_)

## Conclusion

AKI is influenced by a wide range of factors, including patient demographics, preexisting health conditions, specific biomarkers, and external interventions such as surgeries and medications. Recognizing these predictors allows for the implementation of preventive measures, early detection, and timely intervention, potentially mitigating the severity and impact of AKI.

Our study reveals that the presence of comorbidity, High APACHE II score, ventilatory support, use of ionotropic support, and renal replacement therapy were strong predictors of mortality in AKI patients.
